# Supplementary material for: The power of single molecule real-time sequencing technology in the de novo assembly of a eukaryotic genome
Source: Sci Rep. 2015 Nov 30;5:16780. doi: 10.1038/srep16780 (PMC4663752; doi:10.1038/srep16780)
Supplement: Supplementary Information [file srep16780-s1.pdf]

# The power of single molecule real-time sequencing technology in the *de novo* assembly of a eukaryotic genome

Hiroaki Sakai[1], Ken Naito[2]*, Eri Ogiso-Tanaka[2], Yu Takahashi[2], Kohtaro Iseki[2], Chiaki Muto[2], Kazuhito Satou[3], Kuniko Teruya[3], Akino Shiroma[3], Makiko Shimoji[3], Takashi Hirano[3], Takeshi Itoh[1], Akito Kaga[2], and Norihiko Tomooka[2]

[1]Agrogenomics Research Center, National Institute of Agrobiological Sciences, 2-1-2 Kannondai, Tsukuba, Ibaraki, 305-8602, Japan. [2]Genetic Resources Center, National Institute of Agrobiological Sciences, 2-1-2 Kannondai, Tsukuba, Ibaraki, 305-8602, Japan. [3]Okinawa Institute of Advanced Sciences, Uruma, 904-2234, Japan.

Correspondence should be addressed to Ken Naito (knaito@affrc.go.jp).

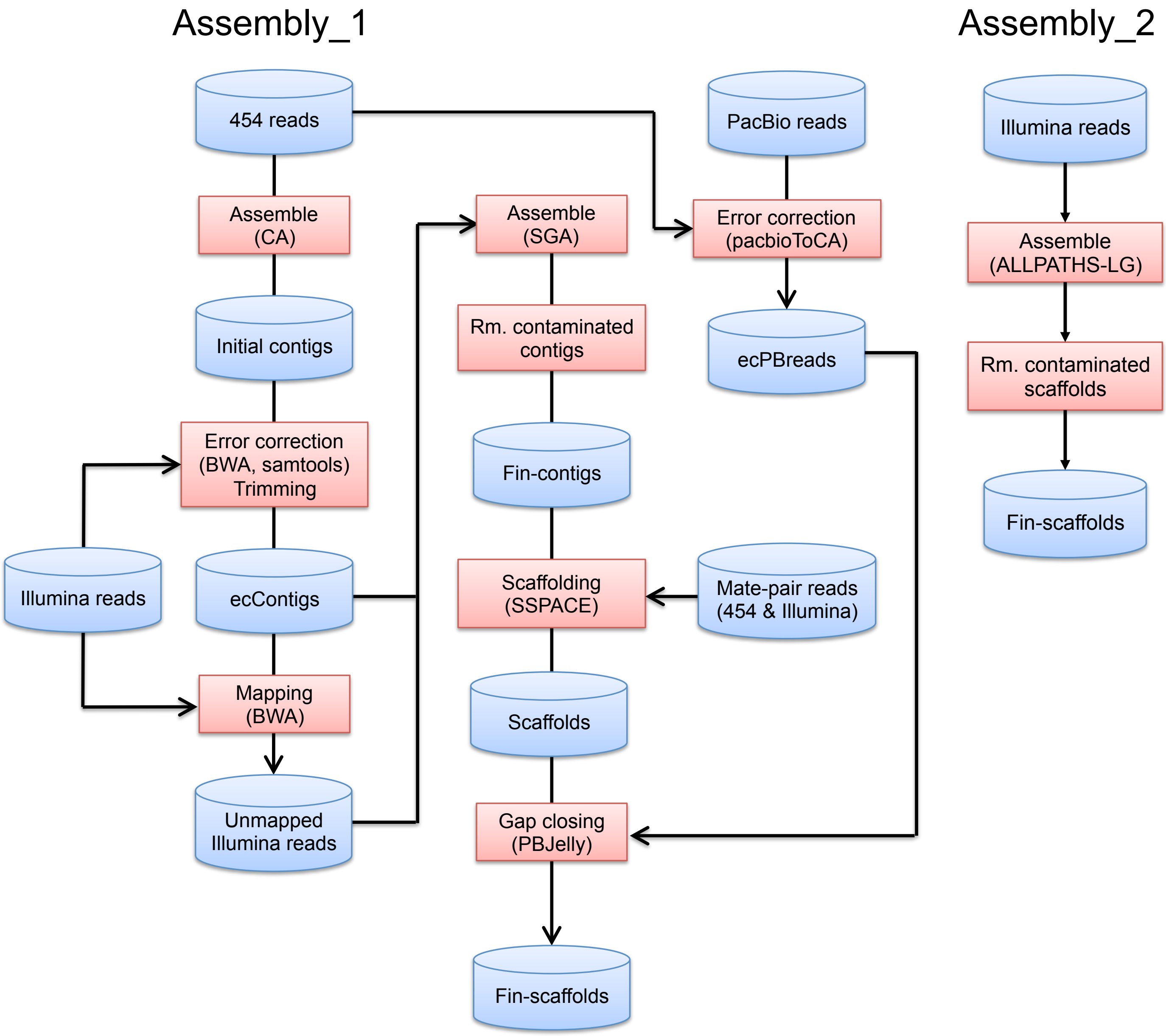


**Supplementary Figure 1. Assembly flows of Assembly_1 and Assembly_2.** Blue drums indicate sequence data, whereas red boxes indicate assembly tools. CA: Celera Assembler. SGA: String Graph Assembler. ecContigs: error-corrected contigs. ecPBreads: error-corrected PacBio reads. Rm: remove. Fin-contigs: final contigs. Fin-scaffolds: final scaffolds.

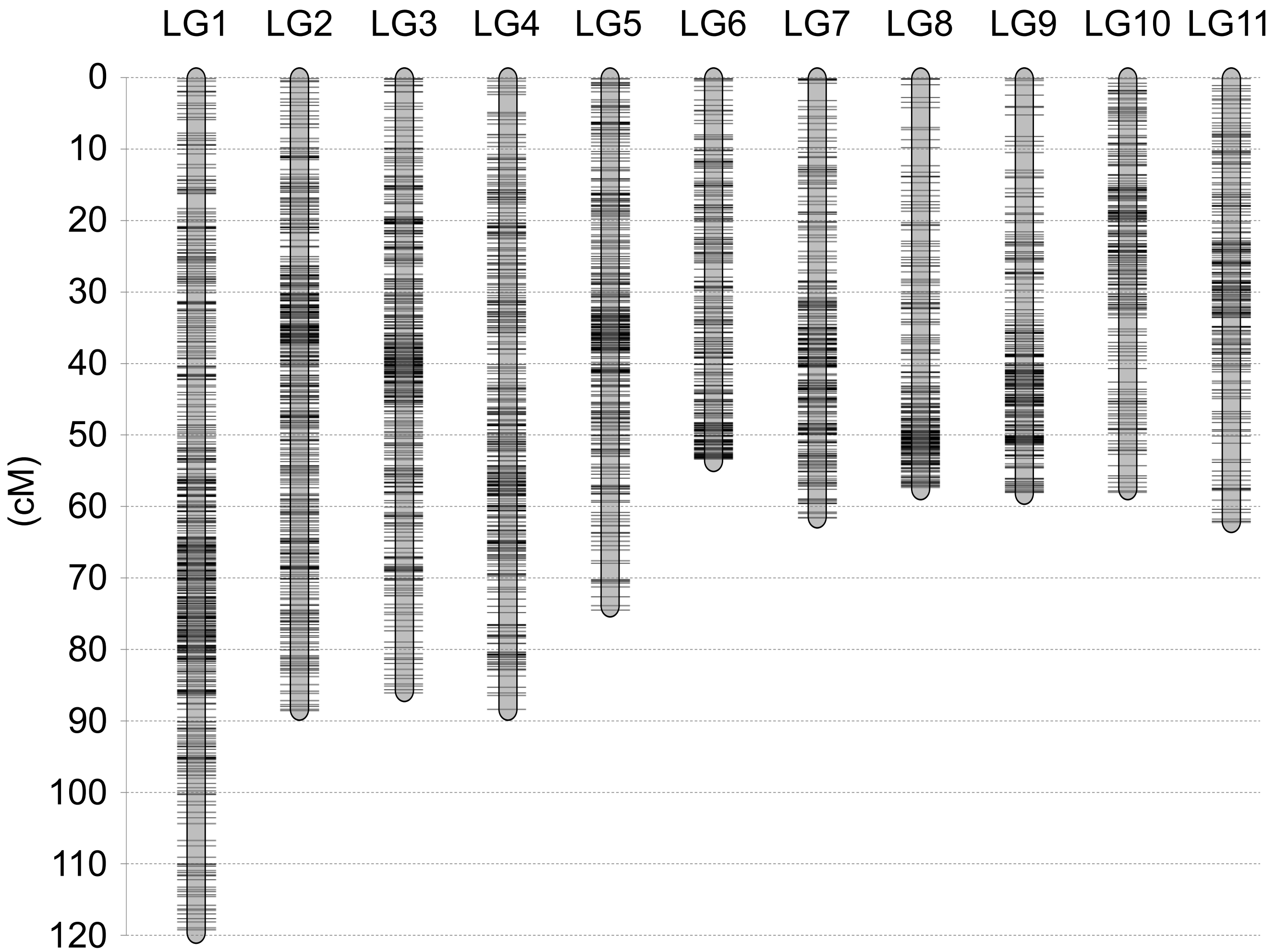


**Supplementary Figure 2. A high-density genetic linkage map of the azuki bean.** In total, 4,912 marker loci were genotyped in 995 F2 plants derived from azuki bean x *V. nepalensis*.

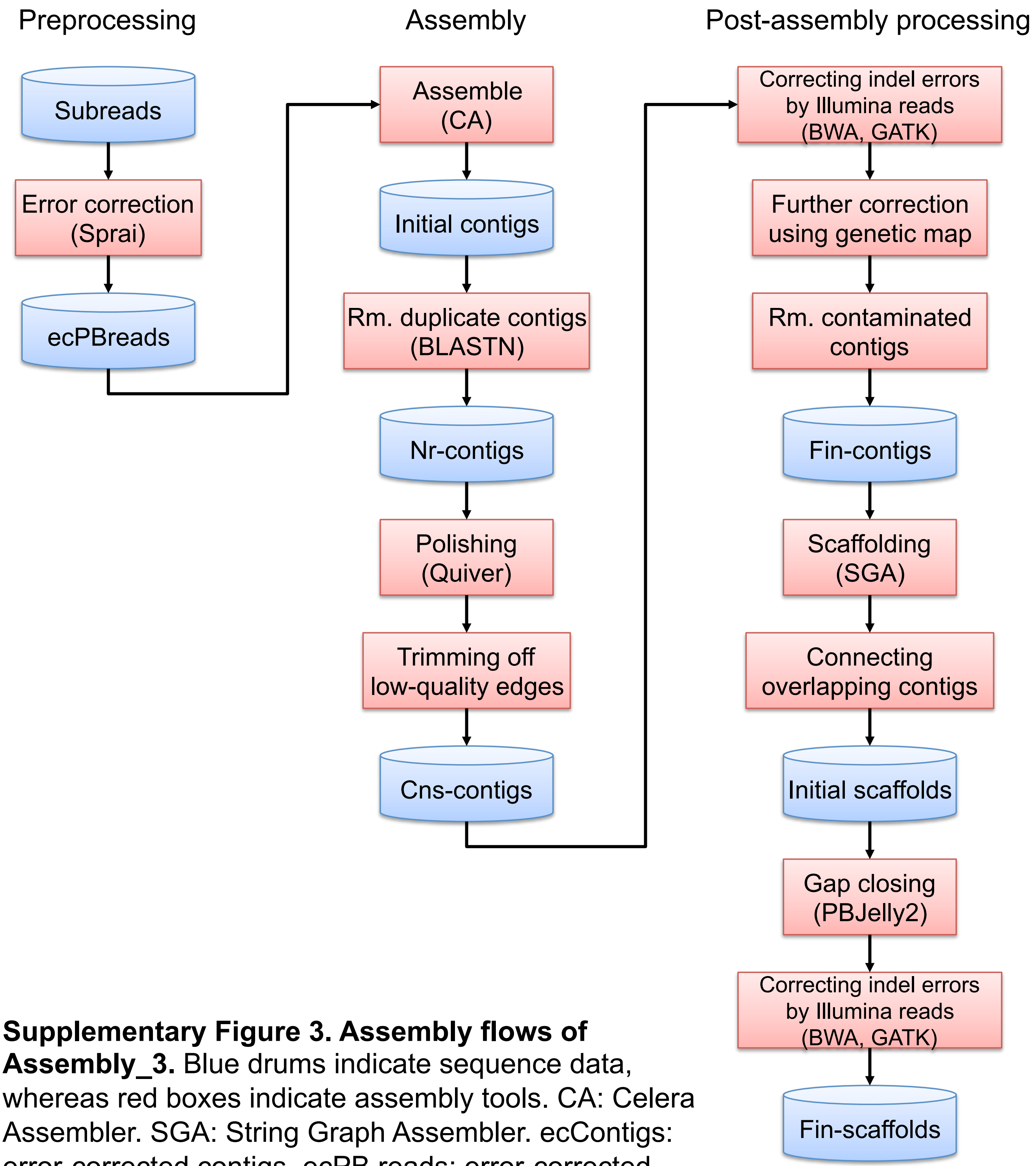


**Supplementary Figure 3. Assembly flows of Assembly_3.** Blue drums indicate sequence data, whereas red boxes indicate assembly tools. CA: Celera Assembler. SGA: String Graph Assembler. ecContigs: error-corrected contigs. ecPB reads: error-corrected PacBio reads. Rm: remove. Nr-contigs: non-redundant contigs. Cns-contigs: consensus contigs. Fin-contigs: final contigs. Fin-scaffolds: final scaffolds.

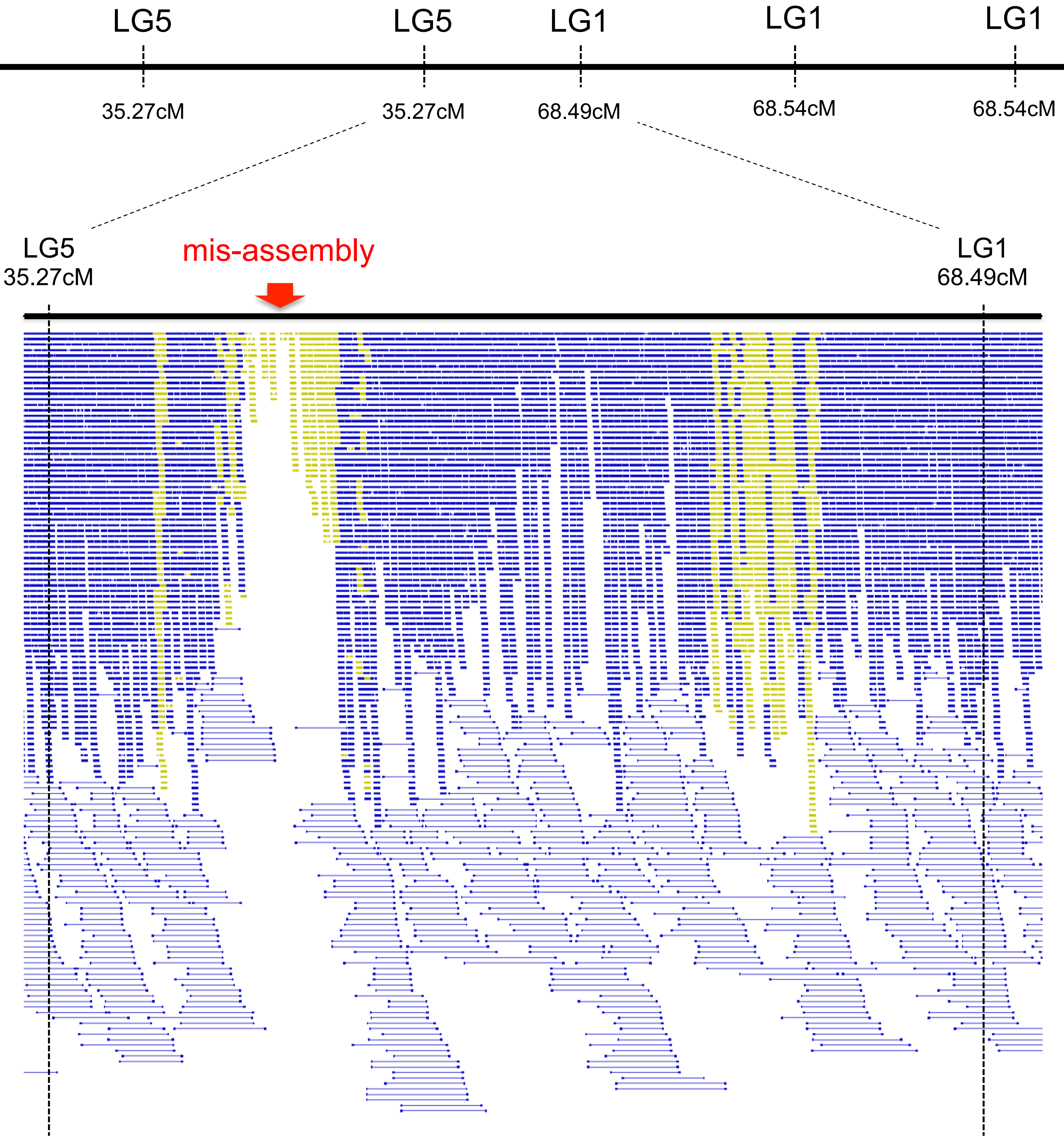


**Supplementary Figure 4. A selection of misassembly sites.** In this contig, DNA markers on LG1 and LG5 were present. Blue thick lines indicate the uniquely mapped paired-end reads with 250 bp inserts 250 bp, while the blue dots bridged with thin lines indicate those of 3 kb inserts. The yellow lines indicate the reads with multiple hits.

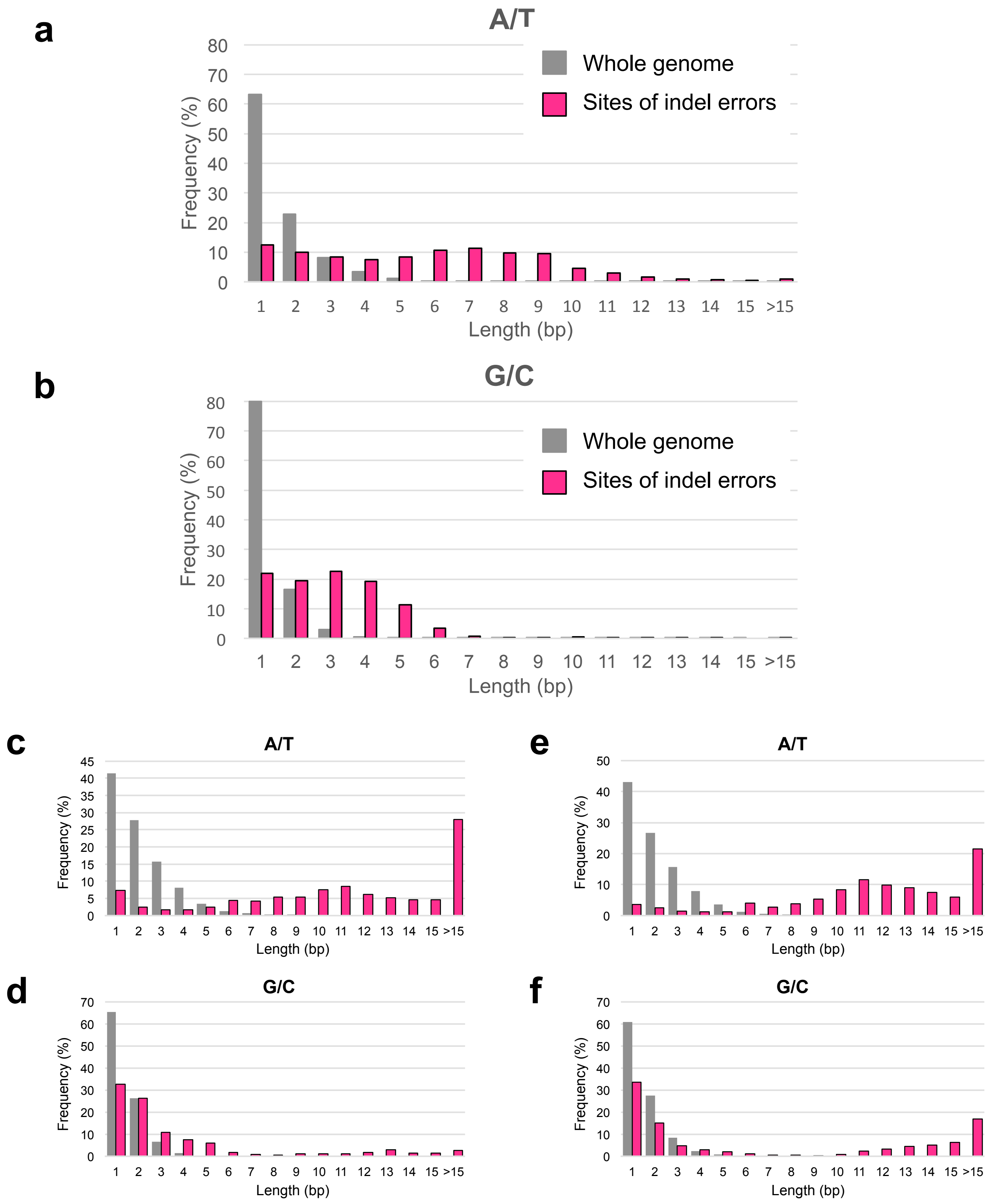


**Supplementary Figure 5. Frequency of homopolymer length at indel-errors in PacBio assemblies. (a)** Homopolymers of A/T or **(b)** Homopolymers of G/C in the azuki bean genome. Gray bars indicate frequency of homopolymers in the whole assembled sequences as a control. **(c)(d)** Arabidopsis thaliana. **(e)(f)** Drosophila melanogaster.

Supplementary Table 3. Summary of pseudomolecules in the Assembly_3.

| | No. of scaffolds | Total scaffolds length | Pseudomolecule length |
|---|---|---|---|
| Chr01 | 35 | 65,919,377 | 67,115,795 |
| Chr02 | 28 | 45,096,540 | 45,515,668 |
| Chr03 | 27 | 41,962,598 | 43,462,759 |
| Chr04 | 25 | 53,470,133 | 54,073,736 |
| Chr05 | 25 | 36,897,451 | 37,380,367 |
| Chr06 | 23 | 38,789,640 | 38,860,970 |
| Chr07 | 20 | 32,020,224 | 33,495,452 |
| Chr08 | 32 | 46,426,616 | 46,951,433 |
| Chr09 | 26 | 35,726,726 | 37,388,052 |
| Chr10 | 17 | 28,785,286 | 28,886,040 |
| Chr11 | 21 | 37,398,860 | 38,115,440 |
| Unmapped | 2250 | 51,515,385 | - |

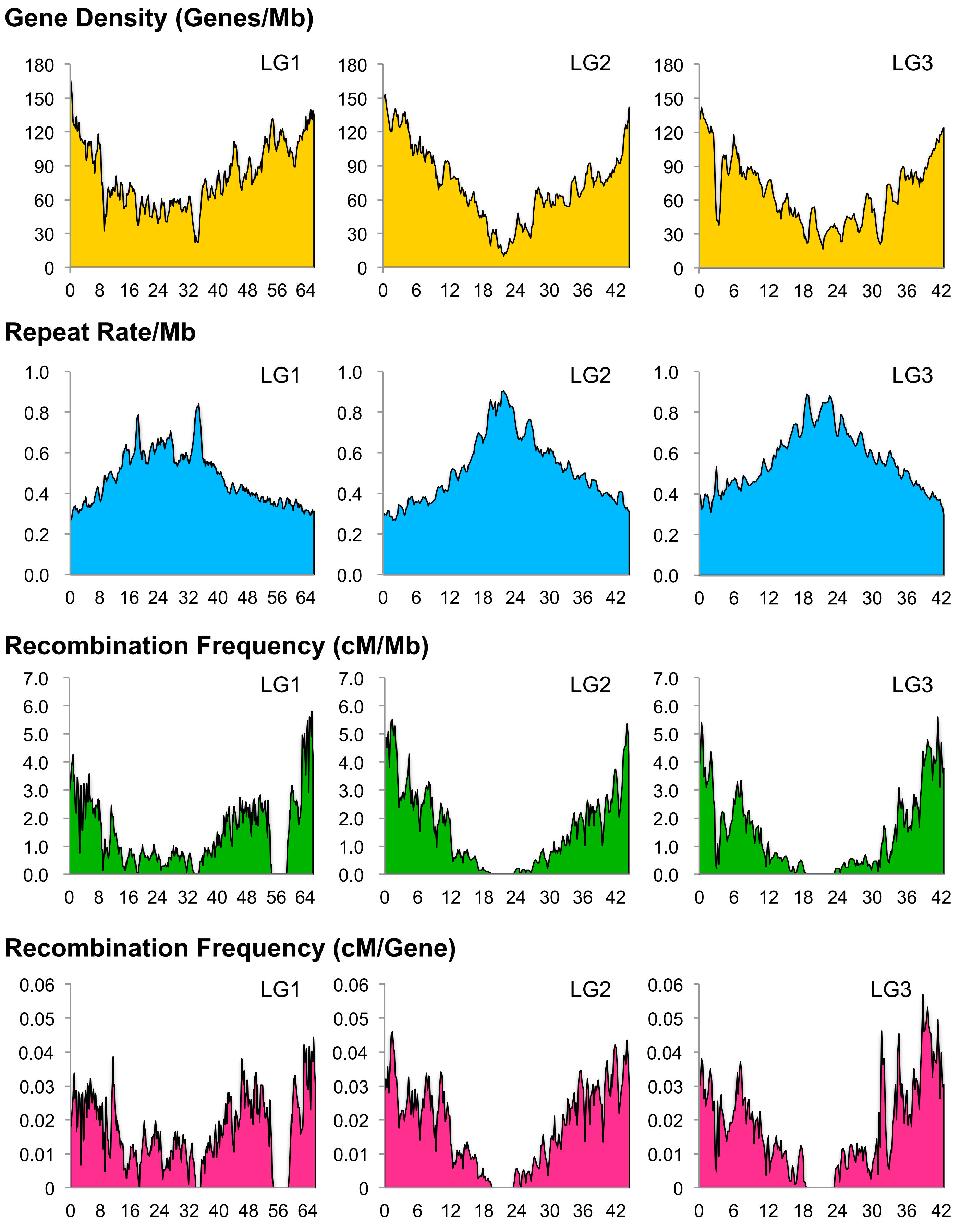


**Supplementary Figure 6. An overview of the azuki bean genome.**

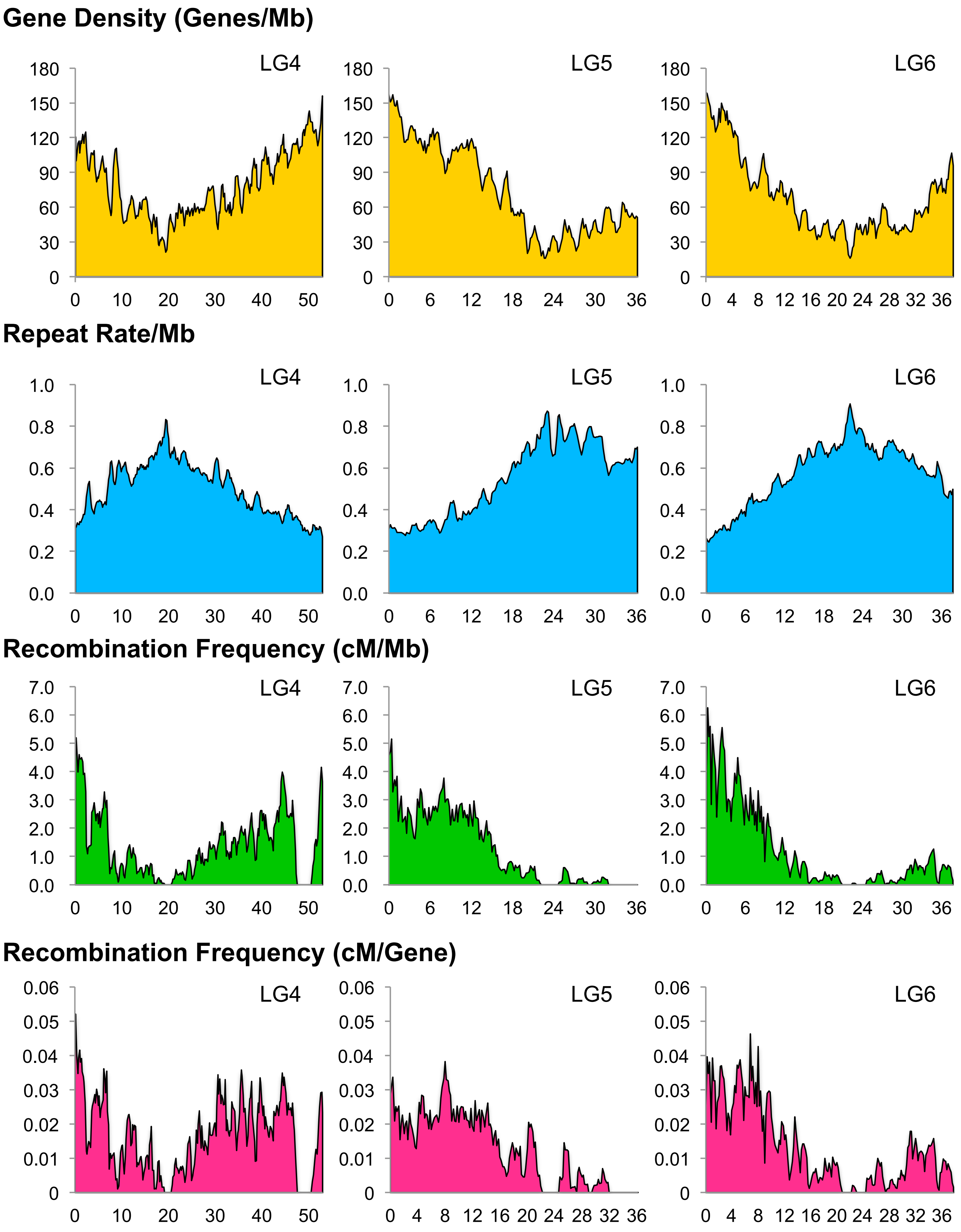


**Supplementary Figure 6. An overview of the azuki bean genome.** Continued.

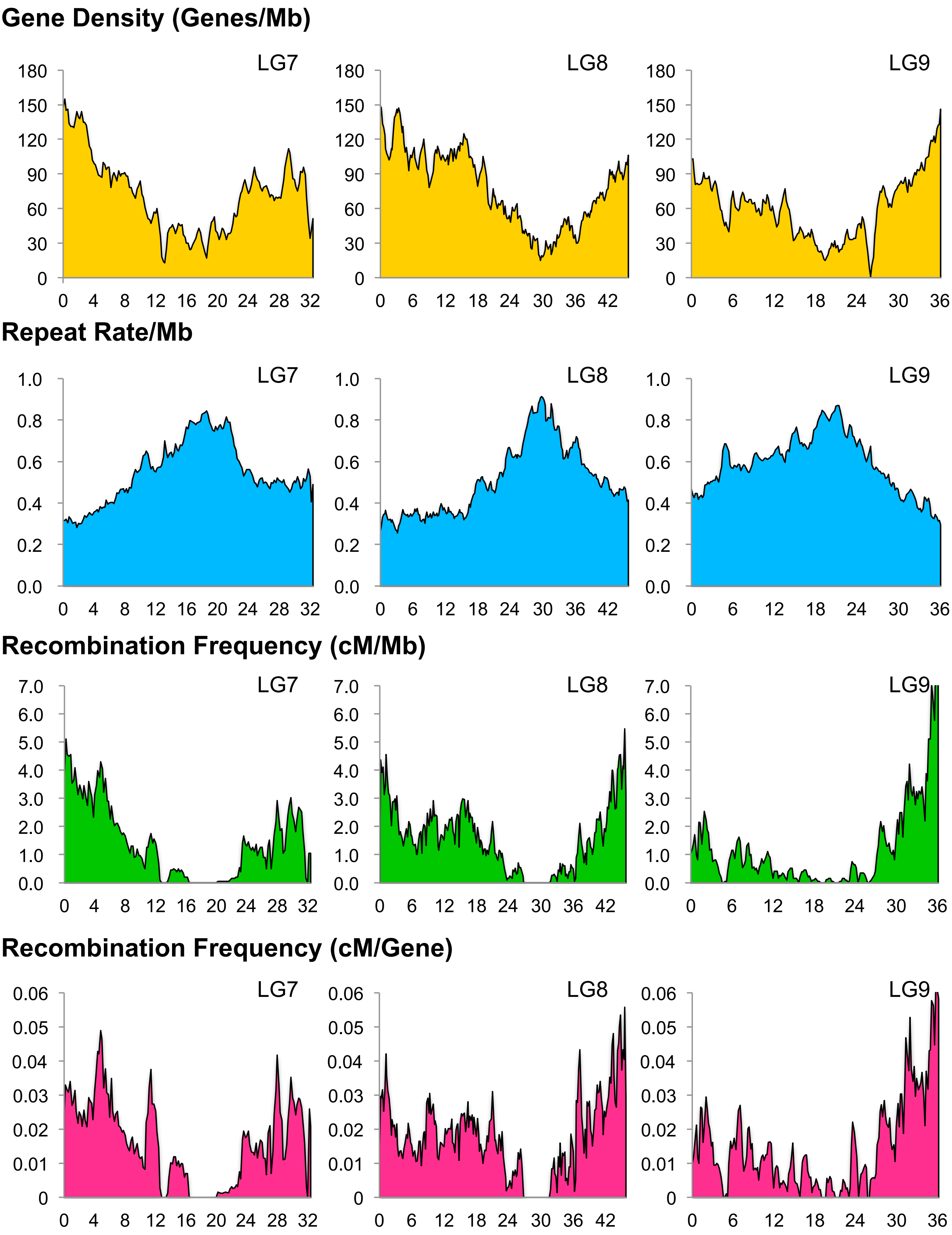


**Supplementary Figure 6. An overview of the azuki bean genome.** Continued.

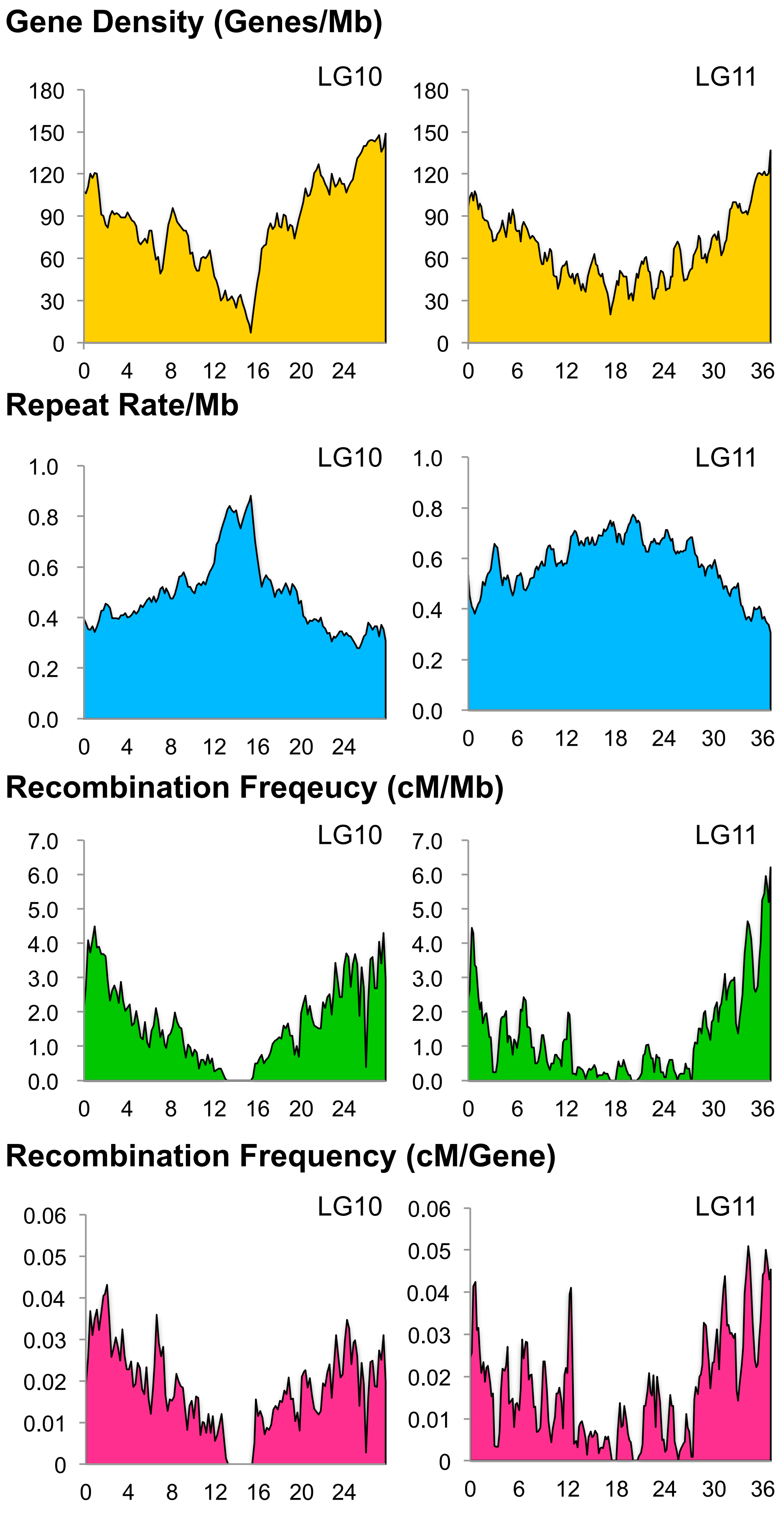


**Supplementary Figure 6. An overview of the azuki bean genome.** Continued.
